# A prediction model for the grade of liver fibrosis using magnetic resonance elastography

**DOI:** 10.1186/s12876-017-0700-z

**Published:** 2017-11-28

**Authors:** Yusuke Mitsuka, Yutaka Midorikawa, Hayato Abe, Naoki Matsumoto, Mitsuhiko Moriyama, Hiroki Haradome, Masahiko Sugitani, Shingo Tsuji, Tadatoshi Takayama

**Affiliations:** 1 Department of Digestive Surgery, Nihon University Faculty of Medicine, 30-1 Oyaguchi Kami-machi, Tokyo, Itabashi-ku 173-8610 Japan; 2 Department of Gastroenterology and Hepatology, Nihon University Faculty of Medicine, 30-1 Oyaguchi Kami-machi, Tokyo, Itabashi-ku 173-8610 Japan; 3 Department of Radiology, Nihon University Faculty of Medicine, 30-1 Oyaguchi Kami-machi, Tokyo, Itabashi-ku 173-8610 Japan; 4 Department of Pathology, Nihon University Faculty of Medicine, 30-1 Oyaguchi Kami-machi, Tokyo, Itabashi-ku 173-8610 Japan; 5 Research Center of Advanced Science and Technology, Genome Science Division, University of Tokyo, 4-6-1 Komaba, Tokyo, Meguro-ku 153-8904 Japan

**Keywords:** Liver fibrosis, Prediction model, Liver stiffness measurement, Magnetic resonance elastography

## Abstract

**Background:**

Liver stiffness measurement (LSM) has recently become available for assessment of liver fibrosis. We aimed to develop a prediction model for liver fibrosis using clinical variables, including LSM.

**Methods:**

We performed a prospective study to compare liver fibrosis grade with fibrosis score. LSM was measured using magnetic resonance elastography in 184 patients that underwent liver resection, and liver fibrosis grade was diagnosed histologically after surgery. Using the prediction model established in the training group, we validated the classification accuracy in the independent test group.

**Results:**

First, we determined a cut-off value for stratifying fibrosis grade using LSM in 122 patients in the training group, and correctly diagnosed fibrosis grades of 62 patients in the test group with a total accuracy of 69.3%. Next, on least absolute shrinkage and selection operator analysis in the training group, LSM (r = 0.687, *P* < 0.001), indocyanine green clearance rate at 15 min (ICGR15) (r = 0.527, *P* < 0.001), platelet count (r = –0.537, *P* < 0.001) were selected as variables for the liver fibrosis prediction model. This prediction model applied to the test group correctly diagnosed 32 of 36 (88.8%) Grade I (F0 and F1) patients, 13 of 18 (72.2%) Grade II (F2 and F3) patients, and 7 of 8 (87.5%) Grade III (F4) patients in the test group, with a total accuracy of 83.8%.

**Conclusions:**

The prediction model based on LSM, ICGR15, and platelet count can accurately and reproducibly predict liver fibrosis grade.

**Electronic supplementary material:**

The online version of this article (10.1186/s12876-017-0700-z) contains supplementary material, which is available to authorized users.

## Background

It is clinically important to diagnose the grade of fibrosis in patients with chronic liver disease. Accurate assessment of liver fibrosis is necessary to determine the indications for treatment of hepatitis C virus infection using direct-acting antivirals [1–3] or interferon therapy [4, 5]. In liver resection, the presence of cirrhosis is associated with blood loss and severe postoperative complications, especially in major liver resection [6]. Therefore, assessment of the extent of fibrosis provides a means to predict surgical risks in patients undergoing liver resection [7].

Percutaneous core-needle biopsy remains the gold standard and has been widely used to evaluate the cause or grade of liver fibrosis in patients with several liver diseases or abnormalities [8]. Although histological diagnosis of biopsy specimens can provide direct diagnostic information, percutaneous liver biopsy is contraindicated in such patients with a tendency to easy bleeding, ascites, or difficult approach for biopsy by ultrasonography.

Recently, liver stiffness measurement (LSM) has become a standard method for assessing liver fibrosis in patients with chronic liver disease [9–12]. Instead of core-needle biopsy, LSM using transient elastography by ultrasound (TE) [9] or magnetic resonance elastography (MRE) [10–12] is a novel, noninvasive, and reproducible method for assessing liver fibrosis to allow treatment. It was reported that LSM using MRE was significantly correlated with the pathological grade of advanced fibrosis in patients with nonalcoholic fatty liver disease [10–12]. Furthermore, LSM by TE was available to predict the risk for liver resection for hepatocellular carcinoma by stratifying the fibrosis of the background liver [13].

This study was performed to establish a prediction model to enable estimation of liver fibrosis based on selected clinical variables, and thereafter confirm the accuracy of our algorithm.

## Methods

### Inclusion and exclusion criteria

Patients that underwent liver resection for malignant tumors in Nihon University Itabashi Hospital from 2014 to 2016 were included in this study. Patients were divided into training and test groups (2:1 ratio). Each participant provided written, informed consent, and this study was approved by the institutional review board of Nihon University (protocol number: RK-141209-4). All clinical investigations were conducted according to the principles of the Declaration of Helsinki.

Patients were included if they were ≥ 20 years of age, candidates for liver resection due to cancer, and provided written informed consent. Patients fulfilling the exclusion criteria were excluded from the study as described previously [14].

### Liver stiffness measurement

MRE was performed as described previously [14]. Briefly, low-frequency (60 Hz) mechanical shear waves with amplitude of 70% were applied to the liver with a proprietary passive driver placed over the right upper quadrant of the abdominal wall for MRE acquisition. After MRE scanning, axial wave and elastogram map images were generated to evaluate quantitative liver stiffness in kilopascals [15, 16].

One radiologist with 10 years of experience, blinded to the histopathology results and all clinical data, measured the liver stiffness by placing the region of interest in the right lobe of the liver on the elastogram maps using the average from four hepatic slice locations.

### Pathology

The degree of fibrosis of resected specimens was determined in accordance with the New Inuyama Classification by two pathologists with more than 5 years of experience in the field of liver pathology [17]. The degree of fibrosis was scored as F0 (no fibrosis), F1 (fibrous portal expansion), F2 (bridging fibrosis), F3 (bridging fibrosis with architectural distortion), or F4 (liver cirrhosis). After pathological diagnosis, the degree of fibrosis was categorized into three grades as follows: no or slight fibrosis, Grade I (containing F0 and F1); moderate fibrosis, Grade II (containing F2 and F3); and liver cirrhosis, Grade III (containing F4).

The degree of necroinflammatory activity was scored as A0 (no necroinflammatory reaction), A1 (mild), A2 (moderate), or A3 (severe). Steatosis was graded according to the Brunt scoring system as follows: 0, none; 1, steatosis in 1%–33% of hepatocytes; 2, steatosis in 33%–66% of hepatocytes; 3, steatosis in 66%–100% of hepatocytes [18].

### Statistical analysis

The correlation coefficient for liver fibrosis was calculated using the Spearman’s rank test. Independent factors for the prediction of liver fibrosis were identified using least absolute shrinkage and selection operator (LASSO) analysis, and the prediction model was established by multiple logistic regression model based on these variables. The predictive ability of fibrosis score was assessed by receiver operating characteristic (ROC) curve analysis and the corresponding area under the curve (AUC).

All statistical analyses were performed using the JMP® 12.0.1 statistical software package (SAS Institute Inc., Cary, NC). In all analyses, *P* < 0.05 was taken to indicate statistical significance.

## Results

### Patients

**Fig. 1:**
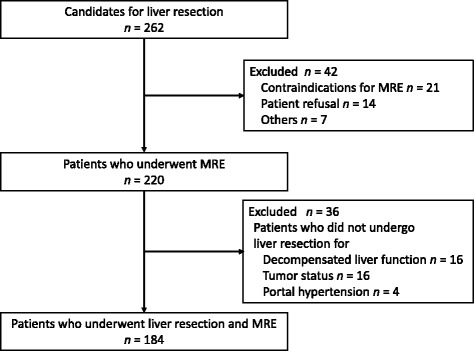
Flow diagram describing patient recruitment and follow-up

The 122 patients enrolled in the first two thirds of the study period were selected for the training group, and the remaining 62 patients in the second one third were selected as the test group (Fig. 1). Eighty-one patients (44.0%) had viral hepatitis. The liver function of 178 patients (96.7%) was Child–Pugh classification A, and the median indocyanine green clearance rate at 15 min (ICGR15) was 12.2% (range: 3.4%–33.0%) (Table 1). The indications for liver resection are summarized in Table 2.

**Table 1:** Patient characteristics

Characteristic	Patients (*n* = 184)
Age, yr	69 (42–91)
Male, n (%)	130 (70.6)
Background liver disease, *n* (%)	
Non-B non-C liver	103 (55.9)
Hepatitis B	35 (19.0)
Hepatitis C	46 (25.0)
Body mass index, kg/m^2^	22.8 (14.4–37.1)
ASA grading, n (%)	
Grade 1	65 (35.3)
Grade 2	116 (63.0)
Grade 3	3 (1.6)
History of hepatic resection, n (%)	48 (26.0)
Hemoglobin, g/dL	13.0 (8.2–16.9)
White blood cell count, 10^9^/L	5.1 (2.3–12.1)
Platelet count, 10^9^/L	17.2 (4.8–43.5)
Total bilirubin, mg/dL	0.59 (0.24–1.48)
Aspartate aminotransferase, U/L	28 (11–170)
Alanine aminotransferase, U/L	23 (6–271)
PT-INR	0.99 (0.85–1.36)
Albumin, g/L	3.9 (2.3–4.8)
Hyaluronic acid, ng/mL	62 (12–737)
IV core 7S, ng/mL	5.8 (3.2–13.0)
Child-Pugh classification, n (%)	
A	178 (96.7)
B	6 (3.2)
ICGR15, %	12.2 (3.4–33.0)
LSM value by MRE, kPa	3.3 (1.4–11.2)

Data were presented as median (range), if not specified

*ASA* indicates American Society of Anesthesiology, *PT-INR* Prothrombin time-International normalized ratio, *ICGR15* Indocyanine green clearance rate at 15 min, *LSM* Liver stiffness measurement, *MRE* Magnetic resonance elastography

**Table 2:** Diseases for liver resection

Disease, *n* (%)	Patients (*n* = 184)
Hepatocellular carcinoma	107 (58.1)
Intrahepatic cholangiocarcinoma	11 (5.9)
Hilar bile duct cancer	4 (2.1)
Gallbladder cancer	4 (2.1)
Cystoadenocarcinoma	1 (0.5)
Metastatic liver cancer^a^	57 (30.9)

^a^From 57 colorectal cancer, two gastric cancer, two pancreatic cancer, and one neuroendocrine tumor

### Pathology of the liver

After the operation, 44 (23.9%), 59 (32.0%), 26 (14.1%), 26 (14.1%), and 29 (15.7%) patients were pathologically diagnosed with liver fibrosis degree F0, F1, F2, F3, and F4, respectively, and 103 (55.9%), 52 (28.2%), and 29 (15.7%) patients were classified into Grade I, Grade II, and Grade III, respectively. Other histological findings including necroinflammatory activity grades and steatosis grades are summarized in Table 3.

**Table 3:** Liver pathology

	Patients (*n* = 184)
Steatosis, n (%)	
0 (0%)	129 (70.1)
1 (1 to 33%)	44 (23.9)
2 (33 to 66%)	10 (5.4)
3 (66 to 100%)	1 (0.5)
Lobular inflammation, n (%)	
A0 (no necro-inflammatory reaction)	41 (22.2)
A1 (mild)	99 (53.8)
A2 (moderate)	43 (23.3)
A3 (severe)	1 (0.5)
Fibrosis, n (%)	
F0 (no fibrosis)	44 (23.9)
F1 (fibrous portal expansion)	59 (32.0)
F2 (bridging fibrosis)	26 (14.1)
F3 (bridging fibrosis with architectural distortion)	26 (14.1)
F4 (liver cirrhosis)	29 (15.7)

### Prediction of liver fibrosis by LSM value

First, we determined a cut-off value for classifying the fibrosis grade using only the LSM value in 122 patients in the training group. The medians of LSM were 0.65 (range, −0.17 to 2.14), 1.25 (0.42 to 3.33), and 2.58 (0.64 to 3.90) in Grade I, Grade II, and Grade III, respectively, in the training group. The cut-off value was determined as the average of the median LSM score of each Grade: Grade I, ≤0.95; Grade II, 0.95–1.91; Grade III, ≥1.91. Using the cut-off value, 26 of 36 (72.2%) Grade I patients, 10 of 18 (55.5%) Grade II patients, and 7 of 8 (87.5%) Grade III patients were correctly diagnosed among the 62 patients in the independent test group, with an accuracy of 69.3% in total.

### Prediction of liver fibrosis by fibrosis score

**Fig. 2:**
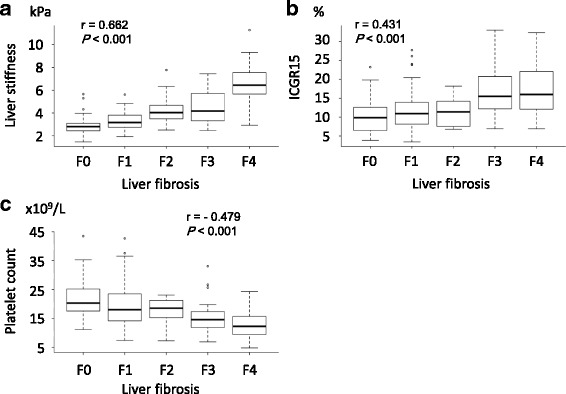
Distribution of LSM by MRE, ICGR15, and platelet count, and liver fibrosis as shown by box plots**.** There were significant correlations between LSM (**a**), ICGR15 (**b**), and platelet count (**c**) and the pathological grade of liver fibrosis

In the training group, liver fibrosis was significantly correlated with LSM value (r = 0.687, *P* < 0.001), ICGR15 (r = 0.527, *P* < 0.001), platelet count (r = –0.537, *P* < 0.001), hyaluronic acid (r = 0.433, *P* < 0.001), and IV core 7S (r = 0.464, *P* < 0.001) on Spearman’s rank test (Fig. 2). On LASSO analysis, LSM, ICGR15, and platelet count were selected as variants for the liver fibrosis prediction model.

Using the three variants, the formula that predicts the fibrosis grade of the liver was established as follows:

Fibrosis Score = 0.428 X_Elast_ + 0.038 X_ICGR15_–0.042 X_Plt_–0.025.

X_Elast,_ LSM value by MRE (kilopascal).

X_ICGR15_, ICGR15 value (%).

X_Plt_, serum platelet count (10^9^/L).

**Fig. 3:**
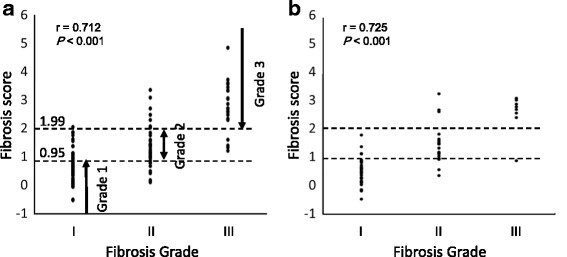
Distributions of fibrosis score and fibrosis grade. **a** Distributions of fibrosis score and fibrosis grade in the 122 patients in the training group (r = 0.712, *P* < 0.001). **b** Distributions of fibrosis score and fibrosis grade in the 62 patients in the test group (r = 0.725, *P* < 0.001)

Based on this prediction model, the median fibrosis scores in patients with Grade I, Grade II, and Grade III in the training group were 0.66 (range, −0.49 to 2.08), 1.24 (0.13 to 3.38), and 2.74 (1.23 to 4.87), respectively (*P* < 0.001). Then, we defined the cut-off value of each Grade as the average of each median fibrosis score in the training group: Grade 1, ≤0.95; Grade 2, 0.95–1.99; and Grade 3, ≥1.99 (Fig. 3a).

Next, we applied this prediction model to the 62 independent patients in the test group, and the median fibrosis scores in patients with Grade I, Grade II, and Grade III were 0.51 (range, −0.45 to 1.84), 1.44 (0.39 to 3.33), and 2.81 (0.92 to 3.15), respectively. According to the definition, 32 of 36 (88.8%) Grade I patients, 13 of 18 (72.2%) Grade II patients, and 7 of 8 (87.5%) Grade III patients were correctly diagnosed in the independent test group, with an accuracy of 83.8% in total (Fig. 3b). The ROC curve for fibrosis grade in relation to the fibrosis score was shown in Additional file 1: Figure S1, and the AUC of the ROC was 0.930 (Fibrosis grade I vs II/III) and 0.925 (Fibrosis grade I/II vs III).

## Discussion

This study showed that the grade of liver fibrosis was noninvasively, accurately, and reproducibly determined using three clinical variables, i.e., LSM, ICGR15, and platelet count. Our prediction model is available to decide the management for patients with chronic liver disease.

There have been several previous reports predicting advanced liver fibrosis using prediction scores combining demographic and clinical routine tests [19–21]. However, as the contribution of LSM by MRE to estimate liver fibrosis was high on multiple logistic regression analyses in this study, a prediction score consisting only of conventional serum markers is considered not to be sufficient for correct diagnosis of liver fibrosis.

Consistent with previous reports, LSM value using MRE was significantly correlated with liver fibrosis in this study. In the distribution of liver fibrosis, the threshold of LSM value was determined using receiver operating characteristic curve analysis between the two groups of liver fibrosis. As a result, the cut-off value of LSM for classifying fibrosis stages 0–2 and 3–4 in nonalcoholic fatty liver disease could be clearly determined [11, 12]. In these reports, MRE-based LSM could predict advanced liver fibrosis, but not cirrhosis, using only MRE-based LSM.

In addition to LSM value, we selected factors contributing to liver fibrosis using LASSO analysis and improved the prediction model containing LSM, ICGR15, and platelet count. Based on these variables, we established a formula to classify chronic liver disease into three grades, and validated the results using an independent patient group. As a result, the accuracy rate of the diagnosis of liver fibrosis grade was remarkably improved compared with the cut-off value by LSM only. Especially, our prediction model has advantages for diagnosis of liver cirrhosis and normal liver.

Our prediction model could predict the normal liver or low grade of fibrosis (F0 and F1) and liver cirrhosis (F4) with high accuracy in the test patient group. On the other hand, the accuracy rate of diagnosis of moderate fibrosis (F2 and F3) by the prediction model was not so high compared with other fibrosis grades. This is because the range of LSM value of F3 stage was wide, which made the prediction of moderate fibrosis difficult. Similarly, LSM by MRE could not clearly divide F1 and F2 in previous reports [11, 12, 22], suggesting that LSM is vulnerable to distinguish the moderate grade of liver fibrosis.

In addition to MRE, LSM using TE is considered the standard method for assessing liver fibrosis [9, 15, 23]. However, only one-directional measurement can be performed in TE including reflection and refraction. In addition, the area measured in the liver using TE is relatively small, which causes sampling variability due to heterogeneity of advanced fibrosis. In contrast, the two-dimensional displacement vector was assessed in MRE [23]. In fact, LSM by MRE was more accurate than that of TE in identification of liver fibrosis [12, 24, 25]. Thus, MRE is more objective, has greater reproducibility, and has better diagnostic accuracy than TE for liver fibrosis.

We usually decide the type of surgical procedure for hepatocellular carcinoma according to liver function, including ICGR15 and serum total bilirubin level [26]. In addition to the diagnosis of liver fibrosis without percutaneous liver biopsy, LSM using TE or MRE could predict the risk of postoperative complications due to blood loss [13]. Furthermore, LSM using MRE could serve as a postoperative predictor of liver regeneration in patients with liver cirrhosis undergoing right hepatectomy [27]. As the surgical outcomes are dependent on massive bleeding during surgery [28] and liver regeneration after resection [29], LSM could provide more accurate decision criteria for selection of the surgical procedure for patients with chronic liver disease.

## Conclusions

We established a model for prediction of liver fibrosis based on ICGR15 and platelet count in addition to LSM by MRE. In contrast to previous reports, our model consists of multiple variables, which could diagnose liver cirrhosis without percutaneous biopsy. It would be available for determination of not only medical treatments but also indications for liver resection.

## Additional file

Additional file 1: Figure S1. ROC analysis for fibrosis score in relation to fibrosis grade. (a) Fibrosis grade I vs II/III. The AUC of the ROC was 0.930. (b) Fibrosis grade I/II vs III. The AUC of the ROC was 0.925. (PPTX 64 kb)

## Abbreviations

AUC: Area under the curve
ICGR15: Indocyanine green clearance rate at 15 min
LASSO: Least absolute shrinkage and selection operator
LSM: Liver stiffness measurement
MRE: Magnetic resonance elastography
ROC: Receiver operating characteristic
TE: Transient elastography by ultrasound
