# Supplementary figures and images for: A prediction model for the grade of liver fibrosis using magnetic resonance elastography
Source: BMC Gastroenterol. 2017 Nov 28;17:133. doi: 10.1186/s12876-017-0700-z (PMC5704624; doi:10.1186/s12876-017-0700-z)

## Slide 1
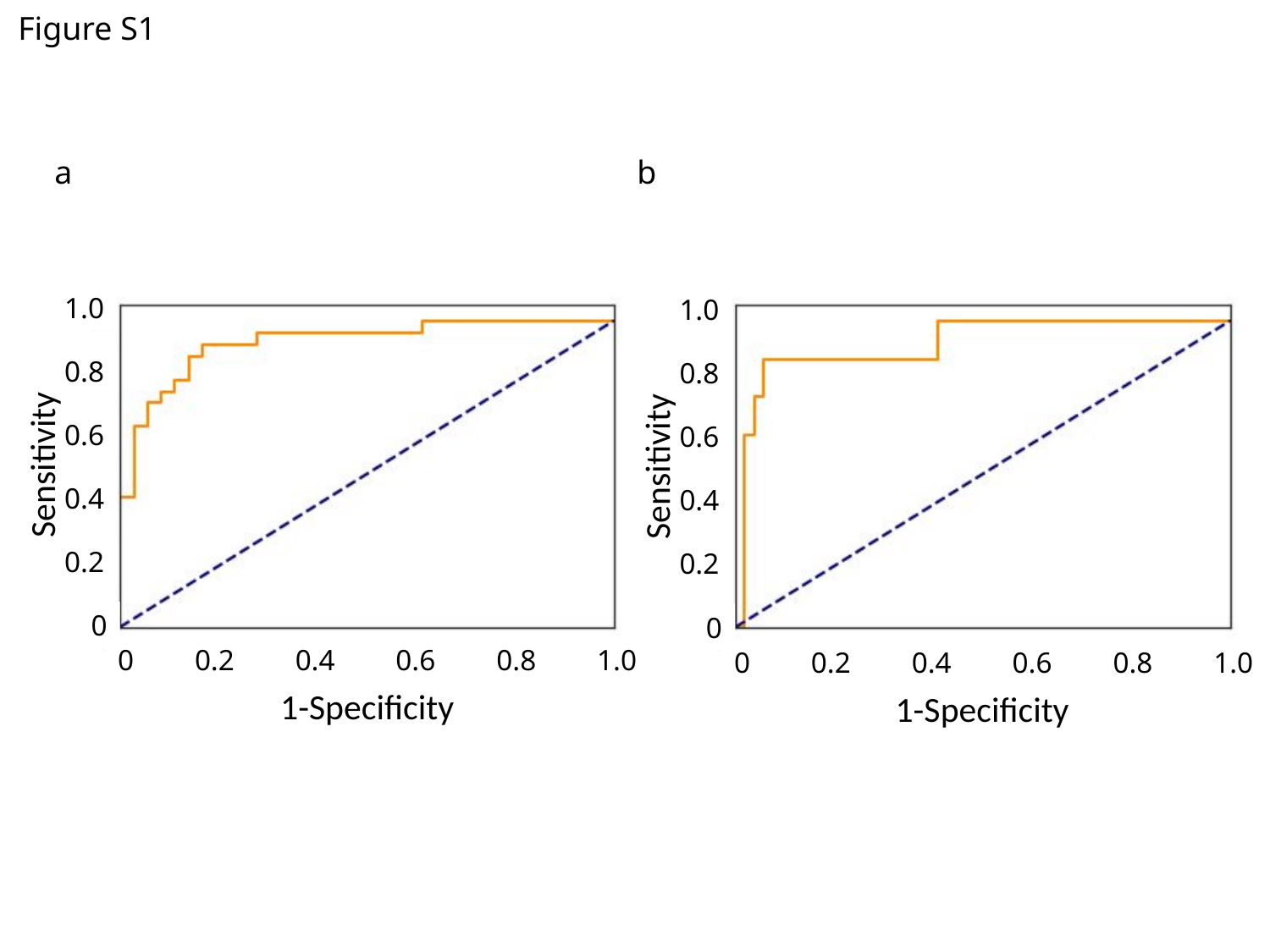

Figure S1
a
b
1.0
1.0
0.8
0.8
0.6
0.6
Sensitivity
Sensitivity
0.4
0.4
0.2
0.2
0
0
0
0.2
0.4
0.6
0.8
1.0
0
0.2
0.4
0.6
0.8
1.0
1-Specificity
1-Specificity

Supplement: Additional file 1: Figure S1. — ROC analysis for fibrosis score in relation to fibrosis grade. (a) Fibrosis grade I vs II/III. The AUC of the ROC was 0.930. (b) Fibrosis grade I/II vs III. The AUC of the ROC was 0.925. (PPTX 64 kb) [file 12876_2017_700_MOESM1_ESM.pptx]
